# Pregnancy- and lactation-induced osteoporosis: a social-media-based survey

**DOI:** 10.1186/s12884-023-05639-w

**Published:** 2023-05-02

**Authors:** Naama Peltz-Sinvani, Hadar Milloh Raz, Pinchas Klein, Sophia Ish-Shalom, Iris Vered, Liana Tripto-Shkolnik

**Affiliations:** 1 Division of Endocrinology, Diabetes and Metabolism, Sheba Medical Center, Tel Hashomer, Ramat Gan, Israel; 2 Sackler School of Medicine, Tel Aviv University, Tel Aviv, Israel; 3 Elisha Hospital, Haifa, Israel

**Keywords:** Osteoporosis, Pregnancy, Lactation, Vertebral fracture, Quality of life

## Abstract

**Background:**

Pregnancy- and lactation-induced osteoporosis (PLO) presenting as spinal fractures is rare, and the spectrum of clinical presentation, risk factors and pathophysiology are incompletely understood. The aim of this study was to delineate clinical parameters, risk factors and osteoporosis-related quality of life (QOL) of women with PLO.

**Methods:**

Participants of a social-media (WhatsApp) PLO group and mothers of a parents’ WhatsApp group (control group) were offered to fill a questionnaire, including an osteoporosis-related QOL section. The groups were compared using the independent Students *t* test for numerical variables, and the Chi-square test or Fisher’s exact test for categorical variables.

**Results:**

Twenty-seven women with PLO and 43 in the control group (aged 36.2 ± 4.7 and 38.8 ± 4.3 years, respectively, *p* = 0.04) participated. Among women with PLO, more than 5 vertebrae were involved in 13 (48%), 4 vertebrae in 6 (22%), and 3 or fewer vertebrae in 8 (30%). Among the 24 women with relevant data, 21 (88%) had nontraumatic fractures; 3 (13%) women had fractures during pregnancy, and the remaining during the early postpartum period. Diagnosis was delayed for over 16 weeks for 11 (41%) women; 16 (67%) received teriparatide. Significantly lower proportions of women in the PLO group engaged in physical activity over 2 hours/week, before and during pregnancy (37 vs. 67%, *p* < 0.015 and 11 vs. 44%, *p* < 0.003, respectively). A lower proportion of the PLO than the control group reported calcium supplementation during pregnancy (7% vs. 30%, *p* = 0.03) and a higher proportion reported treatment with low-molecular-weight-heparin during pregnancy (*p* = 0.03). Eighteen (67%) of the PLO group expressed fear of fractures and 15 (56%) fear of falls, compared to none and 2%, respectively, of the control group (*p* < 0.00001 for both).

**Conclusions:**

Most of the women with PLO who responded to our survey reported spinal fractures involving multiple vertebrae, delayed diagnosis, and treatment with teriparatide. Compared to a control group, they reported less physical activity and impaired QOL. For this uncommon yet severe condition, a multidisciplinary effort should be exerted for early identification and treatment, to alleviate back pain, prevent subsequent fractures and improve QOL.

**Supplementary Information:**

The online version contains supplementary material available at 10.1186/s12884-023-05639-w.

## Introduction

Pregnancy and lactation impose major demands on a woman’s body to supply a proper amount of calcium for the developing fetus and neonate [1]. By term, the fetal skeleton requires about 30 g of calcium, which is supplied by the mother; over 80% of it is consumed during the third trimester [1, 2]. Variable hormonal changes occur to meet this extra demand, resulting in a positive calcium balance during the third trimester [3]. During pregnancy, calcium absorption via the gastrointestinal tract more than doubles, allowing preservation of a mother’s bone density [4, 5]. This process is mediated largely by a kidney and placenta-derived increase in 1,25(OH)2D3 [6, 7]. Other changes, such as increases in prolactin and placental lactogen, which are independent of vitamin D and lead to increased calcium absorption, were demonstrated in animal studies [8, 9]. During lactation, intestinal calcium absorption decreases to its pre-pregnancy level [10]; hence, a high fetal calcium demand is met by maternal bone resorption. This process causes a certain decrease in bone mineral density (BMD), which is usually transient but can temporarily increase fracture risk [11, 12]. A large study published in 2014 showed that prolonged breast-feeding may have a deleterious long-term effect on BMD and may contribute to increased risk of osteoporosis later in life [13].

Both during pregnancy and lactation (mainly during lactation), Parathyroid hormone-related protein (PTHrP), which is secreted from placenta and breasts and stimulate bone resorption via PTH receptor type 1 [12, 14, 15].

Although changes in BMD and bone metabolism during pregnancy and lactation are physiological, a small proportion of women may experience more significant imbalances of calcium and bone metabolism, leading to an adaptation failure [12, 16]. This condition is known as pregnancy- and lactation-induced osteoporosis (PLO) and presents as spinal fractures in late pregnancy or in the early postpartum period. PLO is rare, and its exact incidence is not known. Various sources cite occurrence of about 0.4 in 100,000 [17, 18]. The relatively low incidence may result in delayed diagnosis and treatment [12, 19]. Additional knowledge gaps exist regarding risk factors, the diversity of clinical presentation and the impact of the condition on quality of life (QOL).

In the virtual era, on-line patient support groups via social media, which is unconstrained by temporal and geographical boundaries, has become increasingly popular [20], especially for those coping with rare diseases [21]. This framework may serve as a platform for information gathering and transferring, expert recommendations, experience sharing and emotional support [22].

In the current study we aimed to assess risk factors and clinical parameters related to fractures among women with PLO who paticipated in a social-media support group, and to assess possible risk factors for osteoporosis and osteoporosis-related QOL, compared to a control of women, who participated in a WhatsApp group for mothers of young children.

## Methods

This is a retrospective, self-administered, online questionnaire-based study. Participants of a social-media (WhatsApp) group for women with PLO, which was established by a young woman who was diagnosed with vertebral fractures during pregnancy, were offered to fill a questionnaire, including a specific osteoporosis-related QOL section “Mini-Osteoporosis Quality of Life Questionnaire” (mini-OQLQ) [23]. We used the same WhatsApp platform to create a control group. WhatsApp messaging has become a standard for communication among people with shared interests, e.g. parents of children in school or preschool settings. Thus, the questionnaire was offered to mothers ofyoung children in several WhatsApp-based public preschool classroom chat groups. The questionnaire opens with the statement “provided as part of research “, hence an exemption from signing a letter of consent was granted by the Chaim Sheba Medical Center Helsinki Committee IRB 1917, IORG 1742, FWA 1580, protocol no.7479–20-SMC. The questionnaire included items regarding possible risk factors of PLO, the timing of fractures, methods of diagnosis, and mode of treatment (supplementary material 1). The QOL part of our questionnaire was based on a mini-OQLQ. The latter is a validated [23] self-reported instrument designed to identify functional and emotional difficulties in women with osteoporosis, and with back pain due to vertebral fractures. For this study, the mini-OQLQ questionnaire was translated to Hebrew, according to the guidelines for translating and adapting tests issued by the International Test Commission [24]. The questionnaire requested responders to state their daily consumption of certain foods (milk/ soy drink, hard cheese, yogurt, curd cream cheese/ cottage cheese, raw tahini, ricotta cheese). Considering the minimum consumption of these foods, the responders’ daily dietary calcium intake was calculated according to the online calcium calculator of the Israeli Foundation of Osteoporosis and Bone diseases (a member of the International Osteoporosis Foundation Committee of National Societies) [25].

The study was approved by the institutional review board of Sheba Medical Center and was conducted in accordance with the declaration of Helsinki.

### Statistical analysis

All the data were coded and unified into a common database. Continuous data are expressed as means and standard deviations (SD), or as medians and interquartile ranges. Categorical data are expressed as numbers (n) and percentages. The groups were compared using the independent Students *t* test for numerical variables, and the Chi-square test or Fisher’s exact test for categorical variables. A *p* value of < 0.05 was considered statistically significant.

## Results

Twenty-seven women with PLO and 43 women in the control group were included in the study. Their mean ages were 36.2 ± 4.7 and 38.8 ± 4.3 years, respectively, *p* = 0.04. Their characteristics are presented in Table 1. The groups did not differ significantly in smoking habits, periods of amenorrhea, duration of lactation, corticosteroid use, vitamin D supplementation, or family history of fractures or osteoporosis. The women in the PLO group were diagnosed between a few months to 8 years prior to participation. Women in the control group were mothers of children aged 6 months to 8 years.

**Table 1:** Anthropometric data and clinical features of the respondents to the survey

	PLO	Control	*p* value
n	27	43	
Age (years)- mean (SD)	36.2 (4.7)	38.8 (4.3)	
Smoking before pregnancy	5 (19%)	3 (7%)	0.245
Menstrual irregularities	9 (33%)	19 (44%)	0.455
Physical activity			
Before pregnancy	13 (48%)	31 (72%)	0.074
Before pregnancy ≥2 hours/week	10 (37%)	29 (67%)	**0.015**
During pregnancy	8 (30%)	22 (51%)	0.088
During pregnancy ≥2 hours/week	7 (11%)	19 (44%)	**0.003**
Calcium supplementation during pregnancy	2 (7%)	13 (30%)	**0.034**
Vitamin D3 supplementation during pregnancy	5 (19%)	17 (40%)	0.111
Enoxaparin use	7 (26%)	2 (7%)	**0.038**
Steroid use	2 (7%)	5 (12%)	0.698
Osteoporosis in family	9 (33%)	14 (33%)	1
Fracture in family	1 (4%)	4 (9%)	0.642
Lactation	29 (96%)	37 (86%)	0.236
Dietary calcium intake (mg), mean (SD)	486 (291)	393 (220)	0.17

The data are presented as number, unless stated otherwise

*PLO* pregnancy- and lactation-induced osteoporosis, *SD* standard deviation

Significantly lower proportions of women in the PLO compared to the control group engaged in physical activity for more than 2 hours per week before pregnancy (37% vs. 67%, *p* = 0.015) and during pregnancy (11% vs. 44%, *p* = 0.003).

The mean values of daily dietary calcium intake calculated for the PLO and control groups were 486 ± 291 mg and 393 ± 220 mg, respectively (*p* = 0.17). Two (7%) women in the PLO group recieved calcium supplementation during pregnancy compared to 13 (30%) in the control group (*p* = 0.03). A significantly higher proportion of women with PLO were treated with low-molecular-weight-heparin (LMWH) during pregnancy (26% vs. 7%, *p* = 0.03), and the duration of treatment was longer.

Among the participants with PLO, the mean number of fractures was 3.9. Thirteen (48%) women had fractures of more than 5 vertebrae, 6 (22%) had 4 fractured vertebrae and 8 (30%) had fractures with 3 or fewer vertebrae involved (Table 2). Diagnosis was delayed for more than 16 weeks for 11 (41%) of the women with PLO; 13 (48%) had back pains for over 12 weeks until a diagnosis was made. Twenty-four of the women with PLO responded to items regarding the mechanism and time of fractures, the mode of diagnosis, BMD results and treatment after diagnosis. Of these 24 women, 21 (88%) had nontraumatic fractures; 3 (13%) fractures occurred during pregnancy and the rest during the early postpartum period. Fourteen (58%) of the 24 respondents, reported undergoing an MRI test as part of their evaluation. All 24 underwent a BMD test; only one reported having normal BMD and the rest (96%) reported having low or very low BMD. Twenty-three (96%) were treated with calcium or a vitamin D3 supplement, or both and 16 (67%) reported receiving specific treatment for osteoporosis; teriparatide was the chosen medication for all these patients.

**Table 2:** Information accessed from the women with PLO

The number of vertebral fractures	proportion (%)
> 5	13/27 (48%)
4	6/27 (22%)
3	3/27 (11%)
2	2/27 (7%)
1	3/27 (11%)
**Time until diagnosis**	
≥16 weeks	11/27 (41%)
≥12 weeks	13/27 (48%)
**Nontraumatic fracture** ^a^	21/24 (88%)
**Time of fracture**	
During pregnancy^a^	3/24 (13%)
≤8 weeks postpartum^b^	10/21 (48%)
≤12 weeks postpartum^b^	16/21 (76%)
**Method of diagnosis** ^a^	
MRI	14/24 (58%)
X-ray	6/24 (25%)
CT	4/24 (17%)
**BMD test postpartum** ^a^	24/24 (100%)
**BMD results** ^a^	
Very low	20/24 (83%)
Low and very low	23/24 (96%)
**Treatment after diagnosis** ^a^	
Vitamin D3/Calcium supplementation/Both	23/24 (96%)
Specific treatment for osteoporosis	16/24 (67%)

*PLO* pregnancy- and lactation-induced osteoporosis, *BMD* bone mineral density, *MRI* magnetic resonance imaging, *CT* computed tomography

^a^The numbers and percentiles were calculated out of the 24 women who replied to this section

^b^The numbers and percentiles were calculated out of the 21 women with fractures postpartum

In response to the mini-OQLQ (Table 3), 18 (67%) of the women with PLO expressed fear of fractures and 15 (56%) expressed fear of falls; these proportions compare to none and 1 (2%), respectively, of the control group (*p* < 0.00001 for both). A significantly higher proportion of women with PLO than women in the control group reported some distress or discomfort due to pain (81% vs. 19%, p < 0.00001). Six (22%) of the women with PLO experienced “very much” to “extreme” distress or discomfort due to pain.

**Table 3:** Responses to the quality-of-life questionnaire

	PLO % *n* = 27	Control % *n* = 43	*p* value
**Fear of fracture** (in the last 2 weeks)			
Some of the time ≤	67	0	**< 0.00001**
A good bit of time≤	37	0	**0**
None of the time	11	93	**< 0.00001**
**Fear of falls** (in the last 2 weeks)			
Some of the time ≤	56	2	**< 0.00001**
A good bit of time ≤	48	0	**< 0.00001**
None of the time	19	84	**< 0.00001**
**Distress or discomfort** (in the least 2 weeks)			
Some ≤	81	19	**< 0.00001**
Moderate ≤	74	9	**< 0.00001**
Quite a bit	48	7	**0.0001**
Very much	22	2	**0.011**

*PLO* pregnancy- and lactation-induced osteoporosis

## Discussion

In this study of 27 women with PLO and 43 women in a control group, the majority of women with PLO reported spinal fractures involving multiple vertebrae; the mean number of fractures was 3.9. This is in concordance with other series of women with PLO, which reported mean numbers of fractures per patient of 3.3, 3.8 and 4.2 [26–28]. Worthy of mention, however, is that women with single vertebra involvement might be under-reported. Notably, most of the fractures reported in our survey were nontraumatic, and the diagnosis was often delayed, similar to data of other studies [29, 30]. Most of our respondents with PLO reported vertebral fractures during the early postpartum period, as was described in a comprehansive systematic review [31].

We found that a smaller proportion of women with PLO than women in the control group engaged in physical activity for more than 2 hours per week before or during pregnancy. Several studies have demonstrated that physical activity can result in improved bone strength and decreased fracture risk in children and older adults [32–34]. Thus, it is possible that less physical activity before and during pregnancy might pose a risk for fracture. While a possible bias is that a lower proportion of women in the PLO group may have engaged in physical activity during pregnacy due to PLO-related pain, this should not have influenced the pre-pregnancy levels of activity reported. A significantly smaller proportion of our respondents with PLO were treated with calcium supplementation during pregnancy, compared to controls. Several studies have shown an association of adequate dietary calcium intake, or calcium supplementation during pregnancy and the early postpartum period, with reduced bone resorption [35, 36]. However, a meta-analysis from 2021 showed inconclusive results regarding the effect of calcium supplementation on maternal BMD during mid- to late-pregnancy [37].

A significantly higher proportion of our respondents with than without PLO were treated with LMWH during pregnancy. The effects of LMWH on BMD are controversial with conflicting results [38–41], although it is widely agreed that LMWH is less likely to cause bone loss than unfractionated heparin [41–43]. Preclinical data show an inhibitory effect of LMWH on bone formation compared to the dual effect of unfractionated heparin on both decrease in bone formation and increase in bone resorption [42, 44]. Hence, we speculate that a unique pathophysiology of PLO may lead to higher susceptability to bone insults that are less likely to pose a risk for osteoporosis in the general population.

The respondents with PLO reported significant and profound impairment of quality of life, compared to the control group. This impact on QOL can lead to severe mental distress, and declines in confidence and self-efficacy [45].

We did not observe differences between women with and without PLO in smoking habbits, periods of amenorrhea, lactation duration, steroid use or vitamin D suplementation prior to PLO occurence. Nor did the groups differ regarding family history of fractures or osteoporosis; this contrasts with studies that reported such differences [30, 46, 47].

This study had several limitations that should be considered in the interpretation of the results. It is a retrospective self-administered questionnaire study; hence all clinical details are based on a patient’s self-report, and as such are subjected to a recall bias. Second, it is a small study, based on 27 women with PLO. Consistent with the low frequency of PLO, most studies that addressed this condition included small patient numbers [31]. The largest study to date was an investigation of the subsequent fracture risk of 107 women with PLO [28]. Notably, as our study consisted of a self-reported questionnaire, the possibility of recall bias is relevant. In addition, the study setting, based on a social-media support group, raises the issue of selection bias, as women with a more severe condition may be more likely to seek such communication platform.

In conclusion, our findings suggest that several factors in the peripartum period may pose a risk for PLO, including less physical activity and the use of LMWH, while calcium supplementation might convey a protective effect. The impact of PLO on a young mother’s QOL can be profound and devastating, regarding physical, psychological and social aspects, which are often further aggravated by delays in diagnosis and treatment.

A multidisciplinary effort should be exerted for early identification and treatment of PLO, to alleviate back pain, prevent subsequent fractures and to improve QOL. Additional studies are needed to further elucidate the etiology and pathophysiology of this uncommon yet severe condition, as are studies to appreciate the impact on QOL in this population.

## Supplementary Information

**Additional file 1: Supplementary material 1.** Pregnancy and lactation induced osteoporosis questionnaire.

## Data Availability

The datasets used and/or analyzed during the current study available from the corresponding author on reasonable request.
