# Supplementary material for: Pregnancy- and lactation-induced osteoporosis: a social-media-based survey
Source: BMC Pregnancy Childbirth. 2023 May 2;23:311. doi: 10.1186/s12884-023-05639-w (PMC10152747; doi:10.1186/s12884-023-05639-w)
Supplement: Supplementary file 1 — Additional file 1: Supplementary material 1. Pregnancy and lactation induced osteoporosis questionnaire. [file 12884_2023_5639_MOESM1_ESM.docx]

**Supplementary material 1**

**Pregnancy and lactation induced osteoporosis**

**This questionnaire is presented to you as part of a clinical research, by answering this questionnaire you are giving your consent to participate in this research.**

**For anonymous registration please write your last 4 ID digits.**

1. Age (current)
2. Height (cm)
3. Weight before pregnancy (kg)
4. Weight after pregnancy (kg)
5. Do you smoke?
   1. How many cigarettes per day?
   2. For how many years?
6. Did you smoke before pregnancy?
   1. How many cigarettes per day?
   2. For how many years?
   3. How long before pregnancy did you stop smoking?
      - Less than 3 months before conceiving
      - 3-6 months before conceiving
      - 6-12 months before conceiving
      - Over 12 months before conceiving
7. At what age did you have your first menstruation?
8. Were there periods of menstrual irregularities?
   1. What was the longest period without menstruation? (months)
   2. If there were several periods without menstruation, what was the cumulative time? (months)
9. Were you engaged in physical activity before pregnancy?
   1. What kind of physical activity did you do?
   2. How frequent was your physical activity?
      - ≤1 hour/week
      - ≤2 hour/week
      - ≤3 hour/week
      - ≥4 hour/week
10. Were you engaged in physical activity during pregnancy?
    1. What kind of physical activity did you do?
    2. How frequent was your physical activity?
       - ≤1 hour/week
       - ≤2 hour/week
       - ≤3 hour/week
       - ≥4 hour/week
11. Among the following, which does your daily intake include? (You may choose more than one answer).
    - - Milk/ Soy drink- at least 1 glass
      - Hard cheese- at least 1 slice
      - Yogurt-at least 1 cup
      - Curd Cream cheese/ Cottage cheese- at least 5 spoons
      - Raw Tahini – at least 1 spoon
      - Ricotta cheese- at least 1 spoon
      - Neither
12. Did you take Calcium supplements before pregnancy?
13. Did you take Calcium supplements during pregnancy?
14. Did you take Vitamin D3 before pregnancy?
15. Did you take Vitamin D3 during pregnancy?
16. Have you diagnosed with rheumatologic disorder or inflammatory bowel disease?
17. Have you ever been treated with Clexane?
    1. What was the daily dose?
    2. For how long have you been treated (months)?
18. Have you ever been treated with steroids?
    1. What was the daily dose and the duration (please elaborate if there were different doses during different periods)
    2. In case of steroids treatment only before pregnancy- how long before conception was the treatment stopped? (months)
    3. In case of steroids treatment during pregnancy- how long were you treated? (months)
19. Does someone in your family was diagnosed with Osteoporosis?
    1. Among the following, who was diagnosed with Osteoporosis? (You may choose more than one answer)
       - Sister/Brother
       - Mother/Father
       - Grandmother/Grandfather
       - Aunt (blood related)/ Uncle (blood related)
20. Does someone in your family was diagnosed with fracture not accident related or a fall above standing height?
    1. Among the following, who was diagnosed with fracture not accident related or a fall above standing height? (You may choose more than one answer)
       - Sister/Brother
       - Mother/Father
       - Grandmother/Grandfather
       - Aunt (blood related)/ Uncle (blood related)
21. Did you breastfeed after pregnancy?
    1. For how long did you breastfeed (after all deliveries, cumulative)?
       - 0-3 months
       - 3-6 months
       - 6-12 months
       - 12-24 months
       - ≥ 24 moths
    2. In case of twins, for how long did you breastfeed?
       - 0-3 months
       - 3-6 months
       - 6-12 months
       - 12-24 months
       - ≥ 24 moths
       - I did not breastfeed twins
22. Have you had vertebral fracture/fractures?
    1. How many spines have you broke?
       - 1
       - 2
       - 3
       - 4
       - > 5
    2. For how long have you suffered from back pain until vertebral fracture was diagnosed? (Months)
    3. What was the age you were diagnosed with vertebral fracture?
23. Except for spinal fracture, did you have any other fractures during pregnancy or lactation?
    1. Where was the fracture? (You may choose more than one answer)
       - Arm
       - Forearm
       - Wrist
       - Hip
       - Femoral neck
       - Shin
       - Ankle
       - Rib
    2. What was the age you were diagnosed with non-vertebral fracture?
24. What was the mechanism of the fracture?
    - - Slipped in the bathtub
      - Fell while walking/running
      - Injured while engaging extreme sport (ski, cycling, horse riding…)
      - Motor vehicle accident
      - Injured by heavy object
      - Without any injury (lifting light weight – baby, stroller, shopping bags…)
25. Did the fracture/s occur during pregnancy?
    1. At what week?
26. Did the fracture/s occur after delivery?
    1. How long after delivery did the fracture occur? (weeks)
27. How was/were the fracture/s diagnosed?
    - - CT scan
      - MRI
      - X-ray
      - Based on symptoms (without imaging)
28. Was surgical treatment needed?
29. Was Bone Mineral Density test (DEXA scan) performed?
    1. When was the Bone Mineral Density test performed?
       - During pregnancy
       - After delivery- while breastfeeding
       - After delivery- after completion of breastfeeding/ I did not breastfeed
    2. Do you have the Bone Mineral Density result?
       - What was the Bone Mineral Density result?
         - Normal bone density
         - Low bone density compared to your age group
         - Very low bone density compared to your age group

- Please write according to Bone Mineral Density test, the following data: T-SCORE, Z-SCORE, BMD

1. How were you treated after fracture diagnosis? (You may choose more than one answer)
   - - Calcium supplementation
     - Vitamin D3
     - Specific treatment for osteoporosis (Bisphosphonates/Prolia/Forteo)
2. In case specific treatment for osteoporosis was given, what was the treatment?
   - - Bisphosphonates (Ribon/Actonel/Fosalan/Fosavance/Aclasta)
     - Prolia
     - Forteo
     - I was not treated with specific treatment for osteoporosis
3. How often in the last 2 weeks have you felt afraid of fractures?
   - - All the time
     - Most of the time
     - A good bit of the time
     - Some of the time
     - A little of the time
     - Hardly any of the time
     - None of the time
4. How often in the last 2 weeks have you felt afraid of falling?
   - - All the time
     - Most of the time
     - A good bit of the time
     - Some of the time
     - A little of the time
     - Hardly any of the time
     - None of the time
5. How difficult has it been for you to carry things in the last 2 weeks because of your back problems due to osteoporosis?
   - - Extremely difficult – impossible to do
     - Very difficult – almost impossible
     - Quite a bit difficult
     - Moderately difficult
     - Somewhat difficult
     - Little difficult
     - Not difficult
     - Not applicable
6. How much distress or discomfort have you had because of pain in the last 2 weeks?
   - - Extreme distress or discomfort
     - Very much distress or discomfort
     - Quite a bit of distress or discomfort
     - Moderate distress or discomfort
     - Some distress or discomfort
     - A little distress or discomfort
     - No distress or discomfort
7. How difficult has it been for you to lift things in the last 2 weeks?
   - - Extremely difficult – impossible to do
     - Very difficult – almost impossible
     - Quite a bit difficult
     - Moderately difficult
     - Somewhat difficult
     - Little difficult
     - Not difficult
     - Not applicable
8. How difficult has it been for you to travel in the last 2 weeks?
   - - Extremely difficult – impossible to do
     - Very difficult – almost impossible
     - Quite a bit difficult
     - Moderately difficult
     - Somewhat difficult
     - Little difficult
     - Not difficult
     - Not applicable
9. How difficult has it been for you to do housework in the last 2 weeks?
   - - Extremely difficult – impossible to do
     - Very difficult – almost impossible
     - Quite a bit difficult
     - Moderately difficult
     - Somewhat difficult
     - Little difficult
     - Not difficult
     - Not applicable
10. How difficult has it been for you to take the type of vacation or holiday you enjoy because of your back problems due to osteoporosis?
    - - Extremely difficult – impossible to do
      - Very difficult – almost impossible
      - Quite a bit difficult
      - Moderately difficult
      - Somewhat difficult
      - Little difficult
      - Not difficult
      - Not applicable
11. How much distress or discomfort have you had in the last 2 weeks because it has been painful to stand for a long time?
    - - Extreme distress or discomfort
      - Very much distress or discomfort
      - Quite a bit of distress or discomfort
      - Moderate distress or discomfort
      - Some distress or discomfort
      - A little distress or discomfort
      - No distress or discomfort
12. How difficult has it been for you to vacuum in the last 2 weeks?
    - - Extremely difficult – impossible to do
      - Very difficult – almost impossible
      - Quite a bit difficult
      - Moderately difficult
      - Somewhat difficult
      - Little difficult
      - Not difficult
      - Not applicable
